# Structural Priming and Frequency Effects Interact in Chinese Sentence Comprehension

**DOI:** 10.3389/fpsyg.2016.00045

**Published:** 2016-02-02

**Authors:** Hang Wei, Yanping Dong, Julie E. Boland, Fang Yuan

**Affiliations:** ^1^School of Foreign Studies, Xi'an Jiaotong UniversityXi'an, China; ^2^Center for Linguistics and Applied Linguistics, Guangdong University of Foreign StudiesGuangzhou, China; ^3^Department of Psychology, University of MichiganAnn Arbor, MI, USA

**Keywords:** structural priming, baseline frequency, sentence comprehension, Mandarin Chinese, inverse preference effects

## Abstract

Previous research in several European languages has shown that the language processing system is sensitive to both structural frequency and structural priming effects. However, it is currently not clear whether these two types of effects interact during online sentence comprehension, especially for languages that do not have morphological markings. To explore this issue, the present study investigated the possible interplay between structural priming and frequency effects for sentences containing the Chinese ambiguous construction V NP1 *de* NP2 in a self-paced reading experiment. The sentences were disambiguated to either the more frequent/preferred NP structure or the less frequent VP structure. Each target sentence was preceded by a prime sentence of three possible types: NP primes, VP primes, and neutral primes. When the ambiguous construction V NP1 *de* NP2 was disambiguated to the dispreferred VP structure, participants experienced more processing difficulty following an NP prime relative to following a VP prime or a neutral baseline. When the ambiguity was resolved to the preferred NP structure, prime type had no effect. These results suggest that structural priming in comprehension is modulated by the baseline frequency of alternative structures, with the less frequent structure being more subject to structural priming effects. These results are discussed in the context of the error-based, implicit learning account of structural priming.

## Introduction

The resolution of syntactic ambiguities during online sentence comprehension has been heavily scrutinized for it can provide evidence concerning how people draw on various sources of information. Over the years, researchers have found that factors such as structural simplicity (Rayner et al., 1983), semantic plausibility (Trueswell et al., 1994), and discourse context (Altmann and Steedman, 1988) affect syntactic ambiguity resolution. Another important source of information appears to be the baseline frequency of alternative analyses (Mitchell et al., 1995).

People seem to use baseline frequency information during online sentence processing, which might explain why different languages have different relative clause attachment preferences for sentences such as (1).

(1) Someone stabbed the wife of the football star who was outside the house.

In sentence (1), the relative clause *who was outside the house* may be attached either to *the wife* (high in the tree structure) or to *the football star* (low attachment). Previous studies have shown a preference for high attachment in Spanish, Dutch, and French (Carreiras and Clifton, 1993, 1999; Brysbaert and Mitchell, 1996; Zagar et al., 1997; Traxler et al., 1998), whereas English shows either a weak low-attachment preference or no clear preference (Carreiras and Clifton, 1993, 1999; Traxler et al., 1998). Mitchell et al. (1995) argued that the observed cross-linguistic differences in relative clause attachment can be predicted by the relative frequency of high and low attachment in each language^1^.

These frequency effects represent a long-lasting effect of repeated exposure to syntactic structures in real-life settings, but there is also evidence of repeated exposure effects in experimental settings. Some early studies showed that auditory presentation of many sentences of a particular syntactic structure facilitated processing of subsequent sentences with the same structure (Mehler and Carey, 1967) or affected the interpretation of ambiguous sentences (Carey et al., 1970). More recent work found that repeated exposure to sentences containing a novel or infrequent construction could facilitate comprehension (Kaschak and Glenberg, 2004; Wells et al., 2009) or improve grammaticality ratings (Luka and Barsalou, 2005).

Moreover, there is evidence that recent exposure to as little as one instance of a structure can affect subsequent processing. Such structural priming effects were first observed in English language production (Bock, 1986), but have also been found in comprehension, in multiple languages, and even between languages in bilinguals (e.g., Hartsuiker et al., 2004; Bernolet et al., 2007, 2009). One line of research shows that priming in comprehension is lexically-dependent in that structural priming occurs when prime and target trials have certain lexical overlap but not otherwise (e.g., Branigan et al., 2005; Arai et al., 2007; Traxler and Tooley, 2008; Tooley et al., 2009; Chen, 2010). At the same time, there is evidence that priming in comprehension may occur without verb repetition (Scheepers and Crocker, 2004; Traxler, 2008; Thothathiri and Snedeker, 2008a,b).

More recently, there has been growing evidence of lexically-independent priming effects, both in processing parallel constructions (e.g., *A demanding boss said that a lazy worker did not do the job properly*; Sturt et al., 2010) and in comprehending dative constructions that contain verb anomalies (e.g., *The waitress brunks/exists the book to the monk*; Ivanova et al., 2012). In fact, lexically-independent priming in comprehension appears to have the same strength as priming in production when both were examined within a single experiment that involved identical materials and participants (Tooley and Bock, 2014). Finally, by employing more participants and new analyses methods, Pickering et al. (2013) found both lexically dependent and lexically independent priming effects in comprehending prepositional phrase attachment ambiguities (cf. Branigan et al., 2005). More importantly, these effects persisted, in that they were unaffected by whether prime and target sentences were adjacent or separated by one or two fillers.

The existence of lexically specific and lexically independent priming effects during comprehension are consistent with previous sentence processing research demonstrating frequency-based preferences that are associated with both specific lexical items (Trueswell et al., 1993) and syntactic constructions (Brysbaert and Mitchell, 1996). This consistency in findings suggests that structural priming and frequency-based syntactic preferences may have a common source. Both types of effects stem from experience with language (though at different timescales). Frequency-based preferences have presumably been acquired over a lifetime experience with the language, whereas structural priming effects result from very recent exposure to a single instance of a given structure.

Structural priming effects may interact with frequency-based syntactic preferences, as suggested by several language production studies. For example, a number of Dutch and German studies reported that priming in production may exhibit an *inverse frequency/preference effect*. This refers to the fact that structures that are produced less often seem to exhibit greater priming effects and vice versa (Hartsuiker and Kolk, 1998; Hartsuiker et al., 1999; Hartsuiker and Westenberg, 2000; Scheepers, 2003; Bernolet and Hartsuiker, 2010). The existence of such effects indicates that priming in production is modulated by the baseline frequency of alternative structures.

Recently, Segaert et al. (2011) showed that the preference ratio of two syntactic alternatives is a crucial determinant of structural priming effects in Dutch language production. They measured both the proportion of passive/active picture descriptions following a passive or active prime, and the response latency of the picture description. In Experiment 1, priming with the less frequent passive structure led to an increase in passive picture descriptions. There was no corresponding increase in active picture descriptions following primes of the more frequent active structure, but response latencies were decreased following active primes. In other words, priming increased the frequency of the dispreferred alternative and decreased the response latency of the preferred alternative.

If similar mechanisms are involved in priming in comprehension and production (as suggested in cross-modal structural priming, both from production to comprehension, e.g., Branigan et al., 2005; and from comprehension to production, e.g., Bock et al., 2007), it is expected that priming in comprehension is also modulated by the baseline frequency of alternative structures. Until now, however, comparatively little is known about the possible relationship between structural priming and frequency effects during comprehension.

Although the influence of frequency upon structural priming in comprehension has not been systematically explored, incidental reports from the literature suggest that priming in comprehension is affected by the baseline frequency of alternative analyses. In a visual-world eye-tracking experiment, Scheepers and Crocker (2004) explored whether constituent order ambiguity resolution in German was subject to priming. In German, both subject-verb-object (SVO) and object-verb-subject (OVS) orderings are permitted, though the former is more frequent. Moreover, the German case-marking system is partially ambiguous so that, sentence-initial NPs like “Die Krankenschweser … ” (The nurse [feminine, singular] …) can be interpreted as either subject or object. Scheepers and Crocker found that the kind of constituent order being processed in a prime trial affected the constituent ordering preferences in a target trial. More crucially, relative to the neutral prime condition, only the less frequent OVS structure elicited reliable priming effects, whereas the more frequent SVO structure merely induced a numerical trend. These results suggest the possibility that priming in comprehension is constrained by the baseline frequency, but this conclusion is weakened by the fact that the relevant evidence derives from an experiment conducted for other purposes.

Additionally, note that the existing evidence comes mainly from Germanic languages (English, Dutch, German) in which syntactic functions are marked through morphological variations (in case, gender, number, etc.). The observed priming effects might be elicited by syntactic representations as well as by low-level morphosyntactic features. It is important, therefore, to explore whether structural priming exists, and more importantly, whether it interacts with frequency-based syntactic preferences in languages where syntactic functions are not marked morphologically.

In a recent study, Chen (2010) investigated the online processing of Chinese sentences containing an embedded relative clause (e.g., guma chengzan de biaoge haizai guowai dushu “*The cousin that the aunt praised has been pursuing his study abroad*”). Unlike Germanic languages, Chinese does not have morphosyntactic categories and consequently, syntactic functions are not marked morphologically in this language. This ensures that potential priming effects must rely on the preservation of abstract syntactic representations rather than low-level morphosyntactic features. Using eye-tracking and ERP, Chen found structural priming effects when the verb in the relative clause was repeated across the prime and target sentences but not otherwise (see also Chen et al., 2012, 2013). It seems, therefore, that structural priming in Chinese sentence comprehension is at least partly lexically-dependent.

More recently, Wei (2013) investigated the online processing of temporarily ambiguous Chinese sentences containing the construction V NP1 *de* NP2 (e.g., *baifang zuojia de pengyou* “visit writer de friend”). This construction allows for two possible structural analyses, namely, VP [_VP_V[_NP_[_De__P_NP1 *De*] NP2]] (to visit the writer's friend), and NP [_NP_[_De__P_ [_VP_V NP1] *De*] NP] (a friend who is visiting the writer). These two analyses have identical surface form but distinct underlying syntactic structures. In addition, although this construction allows for two analyses, the relative frequency of the two analyses differs. In the context of V NP1 *de* NP2, over 700 out of 1000 items randomly selected from a corpus ^2^ were used as NP (Zhang et al., 2000).

In a self-paced reading experiment, Wei (2013) found that when the construction V NP1 *de* NP2 was disambiguated to the less frequent VP structure (the VP target condition) there was an effect of prime type: Participants experienced more processing difficulty following an NP prime relative to following either a VP prime or a neutral baseline. In contrast, when the ambiguity was resolved to the more frequent NP structure (the NP target condition), prime type had no effect. These results suggest that structural priming in Chinese sentence comprehension is modulated by frequency-based syntactic preferences, with the less frequent structure being more sensitive to structural priming effects.

However, Wei's (2013) conclusions are weakened by several flaws in the research design. First, each prime sentence was followed by a comprehension question, which might interrupt structural priming. Second, participants were exposed to disproportionate numbers of NP or VP structures in different target conditions. In the NP target condition, there were 24 sentences of the NP structure (6 NP primes plus 18 NP-disambiguated target sentences) but there were only six sentences of the VP structure (6 VP primes). The rate of NP vs. VP structure was 4:1. The opposite holds for the VP target condition. The difference in the base rate of the two structures makes any comparisons between the two target conditions problematic: Having been exposed to an unbalanced number of NP or VP structure, participants might display different processing tendencies across conditions. They might tend to interpret the ambiguous construction V NP1 *de* NP2 as NP in the NP target condition but interpret it as VP in the VP target condition. These different processing tendencies, in turn, might be a confounding variable in the assessment of structural priming effects.

Because of the important theoretical implications of Wei (2013), we reinvestigated the priming of the Chinese ambiguous construction V NP1 *de* NP2 while avoiding these two problems in his research design. First, we removed all comprehension questions following the prime sentences. Second, we added filler sentences of the opposite structure to balance the total number of NP and VP sentences under each target condition (see details in Materials). We expected to replicate the pattern of results from Wei (2013) with the improved design, particularly the interplay between structural priming and baseline frequency in Chinese online sentence comprehension.

## Materials and methods

The purpose of the present experiment was to investigate whether online processing of the ambiguous construction V NP1 *de* NP2 could be affected by the prior presentation of a single prime sentence, and more crucially, whether the strength of structural priming could be modulated by the baseline frequency of alternative structures. In the present study, frequency-based syntactic preference for the construction V NP1 *de* NP2 was determined on the basis of corpus data and sentence-fragment completion data reported in previous research (e.g., Zhang et al., 2000; Hsieh et al., 2009). According to a corpus analysis, the ratio of NP to VP stands at 7:3 (Zhang et al., 2000. See Note 1). The NP advantage was even more distinct in an off-line sentence fragment completion test conducted by Hsieh et al. (2009). Their data showed that the fragment *V NP1 de* was typically continued with a noun phrase, which constituted part of an NP completion 95% of the time (911/960), with VP completions accounting for 5% only. These data make it evident that the initial preference ratio is strongly biased toward the NP analysis. Additionally, in a self-paced reading experiment, Zhang et al. (2000) found that processing difficulty occurred immediately when a semantically equibiased item was disambiguated to the VP analysis but no difficulty occurred when it was disambiguated to the NP analysis, confirming that NP is the preferred analysis for the Chinese reader.

Because syntactic ambiguity resolution has been found to be affected by factors such as semantic plausibility information and discourse context (Van Gompel and Pickering, 2007), we used semantically equibiased items only, thereby holding constant any possible semantic effects. In addition, the construction V NP1 *de* NP2 was placed at the beginning of each sentence, so that its interpretation would not be affected by prior context other than the three types of prime sentences.

### Participants

Fifty-five participants from Xi'an Jiaotong University received a small payment for taking part in the experiment. One participant answered more than 20% of the comprehension questions incorrectly and was excluded from further analyses. The remaining 54 participants answered more than 80% of the comprehension questions correctly, with an average accuracy rate of 94%. Ethical approval for the experiment was granted by the School of Foreign Studies Academic Committee at Xi'an Jiaotong University.

### Materials

The experiment employed a 3 (prime type) × 2 (target type) mixed design, with six experimental lists. Prime type (NP, VP, and neutral primes) was manipulated both within-participants and within-items, and target type (NP- and VP-disambiguated) was between-participants and within-items. The 18 prime sentences and 18 semantically equibiased target sentence pairs were adopted from Wei (2013). See Table 1 for a set of examples. A complete list of prime and target sentences appears in the Supplementary Material.

**Table 1:** **Sample prime and target sentences**.

**PRIME SENTENCES**
(1) weizao zhengju de lvshi yiwei buhui youyen faxian zhenxiang (NP prime) falsify evidence *DE* lawyer think no person find out truth.
*The lawyer who falsified evidence thought that nobody would find it out.*
(2) shusan shanshang de youke zhiwai, tamen qidong jinji yu'an (VP prime) evacuate mountain *DE* tourist besides, they start emergency plan.
*Besides evacuating tourists in the mountain, they started the emergency plan.*
(3) qingwa shi liangqi dongwu, keyi zai ludi he shuizhong shenghuo (Neutral prime) frog is amphibian, can land and water live
*Frogs are amphibians and can live both on land and in water.*
**TARGET SENTENCES**
(1) baifang zuojia de pengyou jianyi zuojia chuangzuo yibu huaju (NP-disambiguated target) visit writer *DE* friend suggest writer write one modern drama
*A friend who was visiting the writer advised him to write a modern drama.*
(2) baifang zuojia de pengyou qijian, XiaoGuo youle xinde xiangfa (VP-disambiguated target) visit writer *DE* friend during, Guo had new idea
*During a visit to the writer's friend, Guo had a new idea.*

The same 18 prime sentences (6 of each type) appeared on all six experimental lists, always immediately preceding a target sentence. Six sentences started with V NP1 *de* NP2 that could only be analyzed as an NP (NP primes), six sentences started with V NP1 *de* NP2 that could only be analyzed as a VP (VP primes), and six sentences of irrelevant structures (neutral primes). The NP and VP primes were created by manipulating the thematic role of NP2. Note that the ambiguity of the construction V NP1 *de* NP2 hinges upon, among other things, the thematic role information associated with NP2. If NP2 can be an agent as well as a theme, then both NP and VP analyses are plausible. If NP2 can only be an agent or a theme, then only the NP or the VP analysis is plausible respectively. This allowed us to construct NP and VP primes by varying the thematic role associated with NP2. Neutral primes started with a subject-predicate construction, which was followed by a second clause commenting on the subject/topic (e.g., qingwa shi liangqi dongwu, keyi zai ludi he shuizhong shenghuo “*Frogs are amphibians and can live both on land and in water*”). Unlike NP and VP primes, these sentences were structurally unrelated to V NP1 *de* NP2, and were considered unlikely to trigger a particular interpretation of the target construction under investigation. Thus, they provide a baseline with which the effectiveness of NP and VP primes can be compared.

Each experimental list included either 18 NP target sentences or 18 VP target sentences. Each target sentence began with a phrase that was semantically equibiased between the NP analysis and the VP analysis. The two conditions differed in the disambiguating region, which was either resolved as an NP or as a VP. There were no lexical or semantic connections between prime and target sentences.

Note that the target sentences were always resolved as the same structure on an experimental list. To balance the total number of NP and VP structures that each participant read, we added 18 filler sentences of the VP structure (VP fillers) to the three experimental lists of the NP target condition, and 18 fillers of the NP structure (NP fillers) to the three experimental lists of the VP target condition. An NP filler started with a V-NP-*de*-NP string that was semantically biased toward the NP analysis (e.g., shuluo haizi de mama “*scold child de mother*”) and was fully disambiguated as NP by the following word (usually a verb, as shown in 2). A VP filler started with a V-NP-*de*-NP string that was semantically biased toward the VP analysis (e.g., caifang jiaoshou de furen “*interview professor de wife*”) and was fully disambiguated as VP by the following word (usually a conjunction or preposition, as shown in 3).

(2) shuluo haizi de mama houhui ziji guande buyan (NP filler)scold child *de* mother regret self discipline not strict*The mother who scolded the child regretted that she had been too lax.*
(3) caifang jiaoshou de furen zhihou, jizhe xiele yipian baodao (VP filler)interview professor *de* wife after, journalist write LE one report*After interviewing the professor's wife, the journalist wrote a report.*

In addition, there were 48 other filler sentences of various structures. None of these fillers contained the construction V NP1 *de* NP2, and they were lexically and semantically unrelated to the experimental sentences. Each participant read 102 sentences in total (18 primes, 18 targets, 18 NP/VP fillers, and 48 other fillers). The experimental sentences and fillers were presented in a single random order across lists, with the constraints that at least one filler sentence intervened between each prime-target pair, and that none of the prime-target pairs was immediately preceded by an NP or VP filler.

To encourage comprehension, some of the filler sentences were followed by a question, including the 18 VP/NP fillers as well as 24 fillers of irrelevant structures. Participants pressed the “Y” or “N” buttons to give their answers and received no feedback. Half of the questions required a “yes” response and half a “no” response.

### Procedure

The experiment was conducted using E-prime software. Participants were tested individually and randomly assigned to an experimental list. They were instructed to read the sentences at a pace that closely approximated their normal reading speed, and to read them carefully so as to answer the questions that followed some of the sentences. Each sentence was presented on a single line, beginning from the left edge of the screen. The first screen that participants saw outlined a sentence using a series of underlines, with each word being covered by a single underline. Participants would then press “Enter” on the keyboard to uncover and read the first word. With each press of the “Enter” button, the next word would be uncovered and the previous word would be covered by an underline again. The construction V NP1 *de* NP2 was presented as one region^3^, followed by the remaining part of the sentences that was presented word by word. Including a practice session of 12 sentences, the whole experiment lasted about 18 min.

### Scoring and data analysis

For each type of target sentence, we analyzed three regions, indicated by slashes “/” and numbers in sentences (4) and (5):

(4) VP-disambiguated target
baifang zuojia de pengyou /qijian_1_,/ XiaoGuo_2_/ youle _3_/
visit writer *DE* friend        during,    Guo           have
xinde xiangfa
new   idea
During a visit to the writer's friend, Guo had a new idea.
(5) NP-disambiguated target
baifang zuojia de pengyou/ jianyi_1_/ zuojia_2_/ chuangzuo_3_/
visit      writer *DE* friend    advise  writer    write
yibu huaju
one modern drama
A friend who was visiting the writer advised him to write a modern drama.

The first region for the statistical analysis was the disambiguating word, which was a conjunction or a preposition in the VP target condition, and a verb in the NP target condition^4^. The second and the third regions corresponded to the post-disambiguation regions. The two post-disambiguation regions were included because self-paced reading task might be subject to spill-over effects, with processing load being carried over from one display to the next (Trueswell et al., 1993; Spivey-Knowlton and Sedivy, 1995). The other regions were not included for further analyses because analyzing those regions did not test any hypothesis-bearing predictions.

Prior to analyzing the data, we eliminated any reading times less than 100 milliseconds (ms) or greater than 2500 ms. This criterion eliminated 0.4% of the data. Next, any reading times that were 3 standard deviations above or below the by-participants condition mean were replaced by the cutoff values (Ratcliff, 1993), which affected 1.8% of the data.

## Results

Table 2 presents mean reading times by region and condition for the experiment. Mean reading times for the disambiguation and the two post-disambiguation regions were subject to 3 (prime type) × 2 (target type) mixed ANOVAs, with separate analyses treating participants and items as random factors. Prime type was treated as a within-participant and within-item factor, and target type was treated as a between-participant and within-item factor.

**Table 2:** **Means and standard deviations in milliseconds for reading times in each region and condition**.

**Target**	**Prime**	**Disambiguation Region**		**Post-disambiguation Region 1**		**Post-disambiguation Region 2**	
		***M***	***SD***	***M***	***SD***	***M***	***SD***
VP-disambiguated	Neutral	508	160	478	123	388	63
	VP	524	156	511	130	386	88
	NP	552	178	565	215	408	83
NP-disambiguated	Neutral	545	147	489	175	420	98
	VP	561	165	436	120	431	132
	NP	540	152	456	96	421	94

### Disambiguation region

In the disambiguation region, there were no effects of prime type or target type, nor an interaction (all *F*s < 1).

### Post-disambiguation region 1

The interaction between prime and target types was significant both by participants and by items^5^ : *F*1_(2, 104)_ = 4.11, *p* = 0.019, *MSE* = 12, 514; *F*2_(2, 68)_ = 4.09, *p* = 0.021, *MSE* = 9285. The effect of prime type was not reliable [*F*1_(1, 52)_ = 1.56, *p* = 0.214; *F*2_(1, 34)_ = 1.39, *p* = 0.255]. The effect of target type approached significance in the by-participants analysis [*F*1_(1, 52)_ = 3.27, *p* = 0.077, *F*2_(1, 34)_ = 2.66, *p* = 0.112], with the less frequent VP-disambiguated targets tending to be read more slowly than the more frequent NP-disambiguated targets. Tests for simple effects showed differences between prime types for the VP-disambiguated targets [*F*1_(2, 104)_ = 4.11, *p* = 0.019; *F*2_(2, 68)_ = 3.83, *p* = 0.027], but not for the NP-disambiguated targets [*F*1_(2, 104)_ = 1.56, *p* = 0.215; *F*2_(2, 68)_ = 1.65, *p* = 0.199].

**Figure 1:**
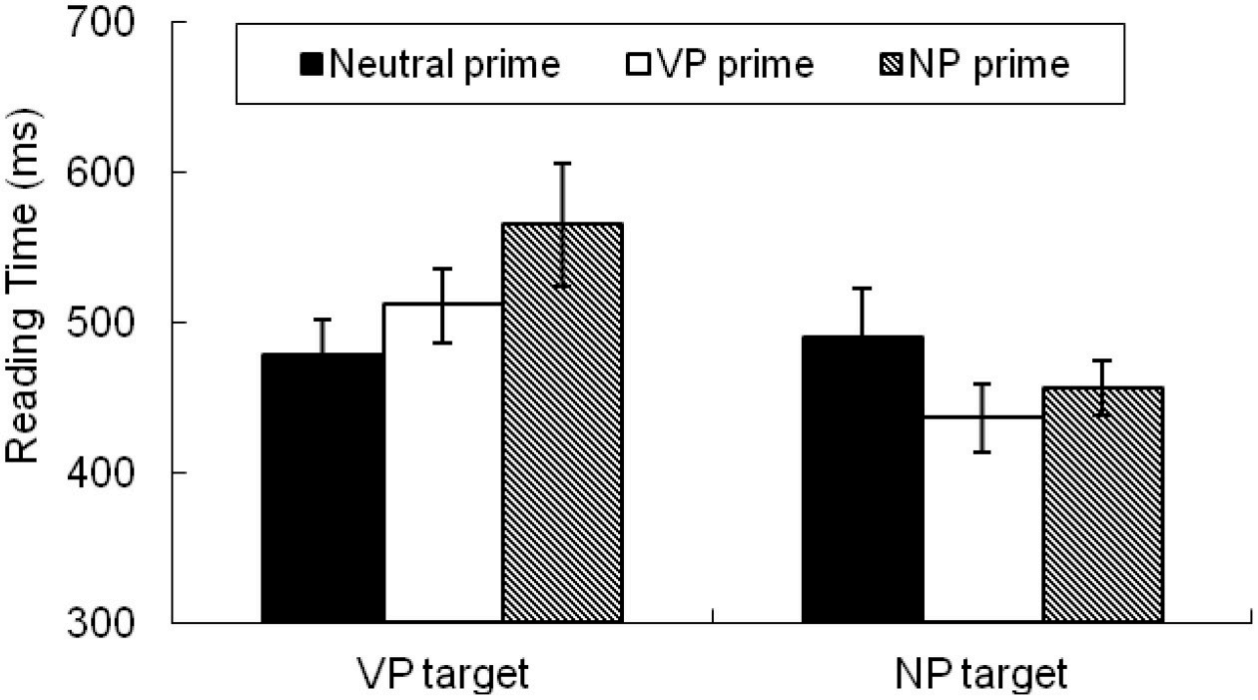
**Mean reading times (with standard errors) by condition for post-disambiguation region 1**.

Figure 1 presents mean reading times by condition for post-disambiguation region 1, which gives a good indication of the variation in processing load associated with syntactic disambiguation as a function of prime and target types. As Figure 1 shows, for the VP-disambiguated targets, the NP prime condition had longer reading times than the Neutral prime condition [565 vs. 478 ms, *t*1_(26)_ = 2.40, *p* = 0.024; *t*2_(17)_ = 2.78, *p* = 0.013]. The NP prime condition also had longer reading times than the VP prime condition, though the difference between them only approached significance in the by-participants analysis [565 vs. 511 ms, *t*1_(26)_ = 1.83, *p* = 0.079] and was not reliable in the by-items analysis [*t*2_(17)_ = 1.22, *p* = 0.239]. The difference between the VP prime condition and the Neutral condition was not reliable [511 vs. 478 ms, *t*1_(26)_ = 1.41, *p* = 0.170; *t*2_(17)_ = 1.15, *p* = 0.268].

### Post-disambiguation region 2

In the second post-disambiguation region, the effect of prime type was not significant (both *F*s < 1), nor was the effect of target type [*F*1_(1, 52)_ = 2.21, *p* = 0.143, *MSE* = 16,803; *F*2_(1, 34)_ = 1.91, *p* = 0.176, *MSE* = 12,521], or the interaction between prime and target, both *F*s < 1.

## Discussion

We investigated the interaction between structural priming and frequency-based syntactic preferences in Chinese sentence comprehension in a self-paced reading experiment. We found that when the temporarily ambiguous target sentence was resolved to the less frequent analysis, participants experienced more processing difficulty (as indicated by the increase in reading times in the first post-disambiguation region) if they had just read a prime sentence of the alternative structure as opposed to a prime sentence of the same structure or a neutral baseline. When the ambiguity was resolved to the more frequent/preferred structure, prime type had no effect on the target sentence.

This pattern of results is similar to that of Wei (2013), though in the present study we eliminated the potential problems existing in Wei's research design (e.g., We removed the comprehension question following each prime sentence and presented participants with equal number of NP and VP structures across conditions. See Materials for details). The current results, paired with those of Wei (2013), suggest that priming in Chinese sentence comprehension is modulated by the baseline frequency of alternative structures, with the less frequent structure being more sensitive to structural priming effects.

The effects of baseline frequency upon structural priming during online sentence comprehension are consistent with previous comprehension studies across several languages (e.g., English, Dutch, French, Spanish), which showed that syntactic ambiguity resolution was affected by the frequency of alternative analyses, with participants preferring more frequent over less frequent ones (see Van Gompel and Pickering, 2007, for a review). Such frequency-based syntactic preferences have presumably been acquired over a lifetime of experience with the language. There has been evidence that repeated exposure to a given structure under experimental conditions produces cumulative priming effects (Kaschak and Glenberg, 2004; Wells et al., 2009). It seems perfectly plausible that repeated exposure to syntactic structures in real-life settings could have similar effects upon the language processing system.

As noted earlier, the effects of frequency seem to be controversial in Chinese relative clause processing. Some studies showed that the more frequent subject relative clauses were easier to process than the less frequent object relative clauses (Kuo and Vasishth, 2006; Lin and Bever, 2006; Liu et al., 2011; Wu et al., 2012), while other studies yielded the opposite results (Hsiao and Gibson, 2003; Chen et al., 2008; Zhang and Yang, 2010; Zhou et al., 2010; Gibson and Wu, 2013; Wang and Bing, 2013). These conflicting results might be partly due to the experimental materials used in these studies. As Wu et al. (2012) noted, most prior studies employed relative clauses that contained two animate NPs (e.g., SRC, raokai bao'an de jizhe “*the reporter that bypassed the guard*,” and ORC, bao'an raokai de jizhe “*the reporter that the guard bypassed*”). This differs from the animacy configurations found in written and spoken corpora (Pu, 2007; Wu, 2009), where the two NPs typically have contrastive animacy configurations, with subject NP being animate and object NP being inanimate (e.g., SRC, duokai shikuai de jizhe “*the reporter that dodged the stone*,” and ORC, jizhe duokai de shikuai “*the stone that the reporter dodged*”). When animacy of the two NPs conformed to this pattern, subject relative clauses were found to be easier to process (Liu et al., 2011; Wu et al., 2012). It seems, therefore, that the baseline frequency also constrains Chinese relative clauses processing, though its effects are modulated by semantic information such as animacy of NPs.

The finding that the less frequent structure was more sensitive to structural priming effects is also consistent with previous research on priming in German sentence comprehension (Scheepers and Crocker, 2004). In their visual-world eye-tracking experiment, Scheepers and Crocker found that the less frequent OVS structure elicited reliable priming effects in German sentence comprehension, whereas the more frequent SVO structure merely induced a numerical but nonsignificant trend. This is similar to what we have found in the present study, though we used a different research paradigm (i.e., self-paced reading) and investigated structural priming in a topologically distinct language (in which alternative structures are not accompanied by morphological variations). Thus, the results of the present study provide cross-linguistic evidence attesting to the constraining effects of baseline frequency upon structural priming in comprehension, and suggest that such effects are not confined to a particular language or research paradigm.

The finding of inverse preference effects in Chinese sentence comprehension has important theoretical implications. There is an ongoing debate over whether structural priming effects are caused by a short-term residual activation mechanism (Pickering and Branigan, 1998), or reflects a form of long-term implicit learning of syntactic structures (Bock and Griffin, 2000; Chang et al., 2006). The finding of such inverse preference effects in comprehension is consistent with the implicit learning account of structural priming, according to which the language processing system learns more about representations that are experienced less often (which follows from error-based learning, a strategy typically adopted in implicit learning algorithms). The presence of such effects appears to be inconsistent with the residual activation account of structural priming, according to which priming originates from transient activation that is sensitive to the immediately preceding structure but may be less affected by the baseline frequency of a given structure.

As mentioned before, the constraints of baseline frequency upon structural priming have also been observed in studies on priming in language production, where structures that are produced less often seem to be more effective in eliciting the target production (Hartsuiker and Kolk, 1998; Scheepers, 2003; Bernolet and Hartsuiker, 2010). However, the inverse preference effects in production appear to hold for response choice, but not necessarily for response time (Segaert et al., 2011; cf. Corley and Scheepers, 2002). In contrast, our inverse preference effect is a type of response time effect, as was the analogous effect observed by Scheepers and Crocker (2004) and Wei (2013). Thus, although structural priming in both comprehension and production appear to be constrained by the baseline frequency of alternative structures, the constraints might manifest differently in production and comprehension.

Note that the priming effects in the present study (as well as in Wei, 2013) occurred in the absence of lexical repetition between prime and target sentences. This stands in contrast to Chen and colleagues' studies (Chen, 2010; Chen et al., 2012, 2013) which showed that priming in Chinese sentence comprehension was crucially dependent upon verb repetition. One possible reason underlying this disparity might be due to the different structures employed in these studies. Chen and colleagues' experimental sentences involved relative clauses, whereas the present study and Wei (2013) employed sentences containing the ambiguous phrase V NP1 *de* NP2. There has been evidence that structural priming in English relative clause occurred only when the same verb was used across the prime and target sentences (Ledoux et al., 2007; Traxler and Tooley, 2008; Tooley et al., 2009), whereas priming in other syntactic structures appeared to be less dependent on lexical repetition (e.g., Traxler, 2008; Ivanova et al., 2012; Pickering et al., 2013; Tooley and Bock, 2014). More investigations should be undertaken to explore this issue.

A final point concerns the finding that relative to the neutral prime condition, VP primes produced no substantial impact on participants' processing of VP-disambiguated targets. This null effect might partly be attributed to the low baseline frequency of the VP structure (Recall that VP accounts for less than 30% in corpus data and only about 5% in sentence completion data; Zhang et al., 2000; Hsieh et al., 2009). As Segaert et al. (2011) suggested, only when the bias against the less preferred syntactic alternative is sufficiently weak a response latency effect prevails. If this reasoning is on the right track, it raises the possibility that a stronger manipulation of the prime conditions would show priming effects of the VP primes.

In sum, the present study provides cross-linguistic evidence that the strength of structural priming during online sentence comprehension is constrained by the baseline frequency of the alternative structures. Particularly, the less frequent structure seems to exhibit greater structural priming effects. The results of the present study, paired with the findings from previous research on other languages, suggest that the constraining effects of baseline frequency upon structural priming (and language comprehension in general) may reflect the operation of some general mechanism that is inherent to the human language processing system.

## Funding

This research was supported by Humanities and Social Sciences Youth Foundation of Chinese Ministry of Education (15YJC740091) and National Social Science Fundation of China (13BYY069).

### Conflict of interest statement

The authors declare that the research was conducted in the absence of any commercial or financial relationships that could be construed as a potential conflict of interest.

## Footnotes

^1^The effects of baseline frequency upon Chinese relative clause processing appear to be controversial. Although subject relative clauses (SRCs, e.g., raokai baoan de jizhe “the reporter that bypassed the guard”) are more frequent than object relative clauses (ORCs, e.g., baoan raokai de jizhe “the reporter that the guard bypassed”) in Mandarin Chinese, prior research has led to conflicting results regarding ease of processing SRCs vs. ORCs (e.g., Hsiao and Gibson, 2003; Lin and Bever, 2006; Chen et al., 2008; Wu et al., 2012; Gibson and Wu, 2013; Wang and Bing, 2013). We will return to this issue in Discussion.

^2^These items were selected from the Corpus for Studies of Modern Chinese (Beijing Language and Culture University, 1995), which contains 1.24 million words covering a wide range of genres.

^3^The primary reason for presenting the construction V NP1 de NP2 as one region was to boost possible priming effects associated with the construction, which might be much weaker if presented word by word. Admittedly, this is not a natural way of reading. However, given that all sentences (including practice sentences, experimental sentences, and fillers) were presented this way, it might not create different expectations among the participants. Debriefing showed that participants were not aware of the structural features of the experimental sentences, nor were they aware of the purpose of the experiment.

^4^The syntactic category difference is unavoidable: To disambiguate the construction V NP1 de NP2 as NP and VP respectively, we had to use words of different syntactic categories. We acknowledge that this weakens comparisons between VP and NP targets, and the possible effect of target type would be difficult to interpret for that reason (we thank an anonymous reviewer for raising this issue). However, the crucial interaction between prime and target is less problematic, because our post - hoc tests showed that it was caused by the prime type differences within each target type (see Results for details).

^5^This pattern of results differs from that of Wei (2013), who found a significant interaction between prime and target types right in the disambiguation region (rather than in the post-disambiguation region). One possible reason for the later occurrence of the expected effects in the present study might have been the use of filler sentences of the opposite structure under the two target conditions (recall that there were 18 filler sentences of the NP structure under the VP target condition, and conversely, there were 18 fillers of the VP structure under the NP target condition. See Materials for details).
