# Supplementary material for: Structural Priming and Frequency Effects Interact in Chinese Sentence Comprehension
Source: Front Psychol. 2016 Feb 2;7:45. doi: 10.3389/fpsyg.2016.00045 (PMC4735403; doi:10.3389/fpsyg.2016.00045)
Supplement: Supplementary file 1 [file DataSheet1.docx]

## Appendix. Prime and target sentences

**Appendix A.1.** Three types of prime sentences (VP primes: 1-6; NP primes: 7-12; neutral primes: 13-18. Slashes indicate presentation regions in the self-paced reading task.)

1. 资助山区的孩子/以来，他们/得到/普遍/好评。

sponsor mountain area DE children ever since, they receive extensive praise

*They have been widely praised ever since they sponsored children in mountain areas.*

2. 看望香港的朋友/期间，老王/患了/甲型/流感。

visit Hong Kong DE friend during, Wang suffer H1N1 flu.

*While visiting his friends in Hong Kong, Wang was infected with H1N1 flu.*

3. 疏散山上的游客/之外，他们/启动/紧急/预案。

evacuate mountain DE tourist besides, they start emergency plan.

*Besides evacuating tourists in the mountain, they started the emergency plan.*

4. 撞伤街上的行人/以后，那个/司机/被/拘留。

run over street DE pedestrian after, that driver BEI arrest

*The driver was arrested after running over a pedestrian.*

5. 寻找现场的证人/同时，他们/继续/深入/调查。

look for scene DE witness while, they continue further investigation.

*While looking for witnesses at the scene, they went on to make further investigation.*

6. 询问当地的居民/之后，事情/终于/真相/大白。

consult local DE residents after, things finally truth clear

*After consulting local residents, the truth finally came out.*

7. 爱好和平的人们/呼吁/政府/停止/战争。

love peace DE people appeal to government stop war

*Peace-loving people appealed to the government to stop war.*

8. 出席会议的代表/表示/就业/压力/仍然/很大。

attend meeting DE representative express employment pressure still intense

*Representatives said at the meeting that employment pressure was still very intense.*

9. 参加考试的学生/反映/试题/难度/有所/增加。

take examination DE student report examination difficulty have increase

*Students who had taken the examination reported it to be more difficult*.

10. 抱怨天气的游客/打算/取消/当天/的/观光/计划。

complain weather DE tourist intend cancel that day DE touring plan

*Tourists who complained about the weather intended to cancel that day’s touring.*

11. 维持秩序的警察/不让/行人/靠近/事故/现场。

keep order DE police not allow pedestrians approach accident scene

*The police that were keeping order prevented pedestrians from approaching the scene of the accident.*

12. 伪造证据的律师/以为/不会/有人/发现/真相。

falsify evidence DE lawyer think no person discover truth.

*The lawyer who falsified evidence thought that nobody would discover it.*

13. 芒果是热带水果，/素有/热带/果王/的/美誉。

mango is tropical fruit, have tropical king of fruit DE reputation

*As a tropical fruit, mangos enjoy the reputation of being the king of tropical fruits.*

14. 月亮是一颗行星，/月亮/自身/其实/不能/发光。

moon is one planet, moon self in fact not give out light

*The moon is a planet which in fact can’t give out light by itself.*

15. 青蛙是两栖动物，/可以/在/陆地/和/水中/生活。

frog is amphibians, can land and water live

*Frogs are amphibians and can live both on land and in water.*

16. 巴西是足球王国，/足球/运动/在/当地/十分/盛行。

Brazil is football kingdom, football in local area very popular.

*Brazil is the kingdom of football where football is very popular.*

17. 熊猫是素食动物，/主要/以/竹子/为食。

panda is vegetarian, mainly take bamboo as food.

*Pandas are vegetarians and live mainly on bamboos.*

18. 中国是茶的故乡，/拥有/丰富/的/饮茶/文化。

China is tea DE hometown, have rich DE tea-drinking culture.

*As the hometown of tea, China has rich tea-drinking culture.*

**Appendix A.2.** Two types of target sentences. In each pair, (a) is NP-disambiguated and (b) is VP-disambiguated. Slashes indicate presentation regions in the self-paced reading task.

1. (a) 拜访作家的朋友/建议/作家/创作/一部/话剧。

visit writer DE friend suggest writer write one modern drama

*A friend who was visiting the writer advised him to write a modern drama.*

(b) 拜访作家的朋友/期间，小郭/有了/新的/想法。

visit writer DE friend while, Guo have new idea

*While visiting the writer’s friend, Guo had a new idea.*

2. (a) 威胁老李的邻居/遭到/大家/的/一致/谴责。

threaten Li DE neighbour receive everyone DE unanimous criticism

*The neighbour who threatened Li was criticized by everyone.*

(b) 威胁老李的邻居/不久，他们/受到/法律/制裁。

threaten Li DE neighbour soon, they receive law punishment

*Not long after having threatened Li’s neighbour, they were punished by law.*

3. (a) 护理丽丽的养父/相信/真情/能够/换来/回报。

nurse Lili’s foster father believe true feelings can exchange repay

*The foster father who nursed Lili believed that one good turn deserves another.*

(b) 护理丽丽的养父/期间，小林/专门/学习/按摩。

nurse Lili’s foster father while, Lin specially learn massage

*While nursing Lili’s foster father, Lin learned massage.*

4. (a) 惦记姑姑的女儿/露出/一副/焦急/的/表情。

be concerned about aunt DE daughter appear one worried DE feelings

*The daughter who was concerned about her aunt appeared very worried.*

(b) 惦记姑姑的女儿/之余，王刚/决定/立即/回家。

worry about aunt DE daughter after, Wang Gang decide at once go home

*Worrying about his aunt’s daughter, Wang Gang decided to go home at once.*

5. (a) 接待小王的同学/详细/介绍/了/社团/情况。

receive Wang DE classmate detailed introduce LE club situation

*The classmate who received Wang made a detailed introduction of the club.*

(b) 接待小王的同学/之前，小李/买了/一些/水果。

receive Wang DE classmate before, Li buy some fruits

*Before receiving Wang’s classmates, Li bought some fruits.*

6. (a) 夸耀自己的球员/输了/那场/重要/的/比赛。

boast about self DE players lose that important DE game

*The players who boasted about themselves lost that important game.*

(b) 夸耀自己的球员/不久，教练/遭到/媒体/非议。

boast about self DE players not long, coach receive media reproach

*Not long after boasting about his players, the coach was reproached by the media.*

7. (a) 督促老吴的雇员/强调/这次/机会/不容/错过。

urge Wu DE employee stress this opportunity not miss

*The employee who urged Wu stressed that this opportunity should not be missed.*

(b) 督促老吴的雇员/以后，效率/有了/明显/提高。

urge Wu DE employee after, efficiency have obviously improve

*There were obvious improvements in efficiency after urging Wu’s employees.*

8. (a) 劝说处长的妻子/认为/大家/的/建议/很好。

Persuade director DE wife think all DE suggestion good

*The wife who persuaded the director thought highly of those suggestions.*

(b) 劝说处长的妻子/之后，事情/开始/有所/好转。

persuade director DE wife after, things begin have good turn

*Things have improved after persuading the director’s wife.*

9. (a) 答复薛明的同事/表示/愿意/提供/技术/支持。

reply to Xue Ming DE colleague agree provide technological support

*The colleague who replied to Xue Ming agreed to provide technological support.*

(b) 答复薛明的同事/之前，小王/广泛/征求/意见。

reply to Xue Ming DE colleague before, Wang extensively solicit opinions

*Before replying to Xue Ming’s colleague, Wang solicited opinions extensively.*

10. (a) 批评团长的参谋/指出/演习/存在/严重/失误。

criticize colonel DE staff officer point out exercise exist serious fault

*The staff officer who criticized the colonel pointed out that there were serious faults in the exercise.*

(b) 批评团长的参谋/同时，首长/作了/自我/检讨。

criticize colonel DE staff officer while, senior officer make self-criticism

*While criticizing the staff officer of the colonel, the senior officer made a self-criticism.*

11. (a) 陪同部长的夫人/表示/这次/出访/进展/顺利。

accompany minister DE wife say the visiting proceed well

*The wife who accompanied the minister said that the visiting went well.*

(b) 陪同部长的夫人/期间，小刘/感到/十分/开心。

accompany minister DE wife during, Liu feel very happy

*Accompanying the minister’s wife, Liu felt very happy.*

12. (a) 讨厌工会的会员/决定/不再/继续/留在/工会。

dislike union DE member decide no longer stay union

*Union members who disliked the union decided to withdraw from it.*

(b) 讨厌工会的会员/之余，小赵/打算/退出/工会。

dislike union DE members while, Zhao decide withdraw union

*Zhao disliked the union members and decided to withdraw from the union.*

13. (a) 嫉妒张明的同桌/故意/制造/了/一些/麻烦。

envy Zhang Ming DE deskmate on purpose cause LE some trouble

*The deskmate who envied Zhang Ming caused a lot of trouble.*

(b) 嫉妒张明的同桌/之余，小华/决心/努力/学习。

envy Zhang Ming DE deskmate while, Hua decide hard study

*Envying Zhang Ming’s deskmate, Hua decided to study hard.*

14. (a) 关心学校的老师/认为/不能/盲目/扩大/招生。

care about school DE teacher think not blindly enlarge enrolment

*Teachers who care about the school hold that enrolment should not be enlarged blindly.*

(b) 关心学校的老师/以外，他们/还/资助/贫困/学生。

care about school DE teacher besides, they also sponsor poor student

*Besides caring about teachers of the school, they also sponsored poor students.*

15. (a) 警告老张的司机/强调/酒后/驾驶/非常/危险。

warn Zhang DE driver stress after-drinking driving very dangerous

*The driver who warned Zhang stressed that driving after drinking was very dangerous.*

(b) 警告老张的司机/之前，警方/接到/了/投诉/电话。

warn Zhang DE driver before, police receive LE complaint call

*Before warning Zhang’s driver, the policeman had received call of complaint.*

16. (a) 称赞曹娟的老师/号召/大家/向她/学习。

praise Cao Juan DE teacher call on everybody from her learn

*The teacher who praised Cao Juan called on everyone to learn from her.*

(b) 称赞曹娟的老师/之后，校长/转身/走出/教室。

praise Cao Juan DE teacher after, president turn walk out of classroom

*After praising Cao Juan’s teacher, the president left the classroom.*

17. (a) 惊动娟娟的小狗/衔着/一只/鞋子/满地/乱跑。

startle Juan DE puppy hold in the mouth one shoe everywhere run

*The puppy that startled Juan held one shoe in the mouth and ran everywhere.*

(b) 惊动娟娟的小狗/以后，小赵/赶紧/躲在/门后。

startle Juan DE puppy after, Zhao quickly hide behind a door

*After startling Juan’s dog, Zhao quickly hid behind a door.*

18. (a) 打击日军的炮兵/受到/总部/的/特别/表彰。

attack the Japanese army DE artillery receive headquarters DE special commendation

*The artillery that attacked the Japanese army was commended by the headquarters.*

(b) 打击日军的炮兵/同时，他们/策划/全面/进攻。

attack the Japanese army DE artillery while, they plan overall attack

*While attacking the Japanese artillery, they were planning an overall attack.*
